# *PIPE-chipSAD:* A Pipeline for the Analysis of High Density Arrays of Bacterial Transcriptomes

**DOI:** 10.3389/fmolb.2016.00082

**Published:** 2016-12-20

**Authors:** Silvia Bottini, Elena Del Tordello, Luca Fagnocchi, Claudio Donati, Alessandro Muzzi

**Affiliations:** ^1^GSK Vaccines SrlSiena, Italy; ^2^Computational Biology Unit, Research and Innovation Centre, Fondazione Edmund MachSan Michele all'Adige, Italy

**Keywords:** high density arrays, tiling arrays, microarrays, transcriptomes, code:python

## Abstract

*PIPE-chipSAD* is a pipeline for bacterial transcriptome studies based on high-density microarray experiments. The main algorithm *chipSAD*, integrates the analysis of the hybridization signal with the genomic position of probes and identifies portions of the genome transcribing for mRNAs. The pipeline includes a procedure, *align-chipSAD*, to build a multiple alignment of transcripts originating in the same locus in multiple experiments and provides a method to compare mRNA expression across different conditions. Finally, the pipeline includes *anno-chipSAD* a method to annotate the detected transcripts in comparison to the genome annotation. Overall, our pipeline allows transcriptional profile analysis of both coding and non-coding portions of the chromosome in a single framework. Importantly, due to its versatile characteristics, it will be of wide applicability to analyse, not only microarray signals, but also data from other high throughput technologies such as RNA-sequencing. The current *PIPE-chipSAD* implementation is written in Python programming language and is freely available at https://github.com/silviamicroarray/chipSAD.

## Introduction

The rapid development of new and high-throughput technologies to conduct genome-wide studies has dramatically increased the ability to discover new non-coding regulatory RNAs. Among the several transcriptome-profiling methods (Zhang et al., 2014), high-density DNA tiling microarrays (Selinger et al., 2000) have been successfully applied to a variety of transcriptome studies (Aparicio et al., 2004; Bertone et al., 2004; Weber et al., 2005; Crawford et al., 2006; Liu, 2007; Heidenblad et al., 2008).

The analysis and interpretation of high density microarray data is based on a precise definition of discrete transcriptional units, thus requiring a specific algorithm to identify them. In particular, the main challenge is to segment the hybridization signal along the genomic coordinate to accurately obtain transcripts boundaries, especially when also non-coding regions are probed. Different statistical algorithms have been developed to process high-density array data and to obtain such segmentation. The widest exploited method was introduced by Kampa et al. (Kampa et al., 2004) and was successively implemented in the Tiling Array Software (TAS) (http://www.affymetrix.com/estore/partners_programs/programs/developer/TilingArrayTools/index.affx). The method was based on the generation of a transcription map constructed by collecting neighbor expressed probes, i.e., probes with a smoothed intensity above a given threshold. TAS extended the method of Kampa et al. by estimating the significance of differential expression using a Wilcoxon signed-rank test within local windows of a given width, centered on each probe. More recently, the Model-based Analysis of Tiling-arrays (MAT) was introduced by Johnson et al. (Johnson et al., 2006). MAT standardized the probe signal value through a model, eliminating the need for sample normalization. MAT used an innovative function specifically designed to score regions of chromatin immunoprecipitation (ChIP) enrichment, which allowed robust *p*-value and false discovery rate calculations. However, both methods were not able to detect short transcripts. A solution to the segmentation problem was also proposed by Huber et al. (Huber et al., 2006) they used a change point detection algorithm, based on a dynamic programming approach, that determined the global maximum of the log-likelihood of a piecewise constant model. This model provides good performances, but it doesn't take the probes position into account, making data analysis less accurate in partially covered genomes. Finally, Thomassen et al. (Thomassen et al., 2009) described a new approach to address the problem of segmentation using a sliding and expanding window running along the genomic coordinate. However, the size of the windows employed by Thomassen et al. could assume only three values making the approach narrowly applicable.

Despite the availability of all these algorithms (Kampa et al., 2004; Huber et al., 2006; Johnson et al., 2006; Thomassen et al., 2009), a comprehensive available pipeline for high-density array data analysis is still lacking, in particular for bacterial transcriptomes for which an increasing amount of genome annotations are becoming more and more accessible (Land et al., 2015; Loman and Pallen, 2015). Henceforth, at our knowledge, no existing tool is able to analyse more than one experiment at the same time especially if also the non-coding portions of the genomes are probed.

To address these needs, we have developed *PIPE-chipSAD*, a pipeline to conduct high-density array data analysis. The main algorithm is *chipSAD* (chip Signal Areas Detector), which provides the segmentation of the hybridization signal and defines the boundaries of the detected transcripts. *Anno-chipSAD* performs an annotation of these regions guiding to the identification of new architectural features as operons, small-RNAs and antisense messenger RNAs. Finally, *align-chipSAD* identifies the transcriptional units analyzing multiple experiments from different chip layouts at the same time.

Herein we provide explications of the main steps of *PIPE-chipSAD* and details of the algorithms strategies. We also show the application of *PIPE-chipSAD* on two previously published datasets (Mellin et al., 2010; Fagnocchi et al., 2015) used to study the transcriptome variation of *Neisseria meningitidis* Δ*hfq* mutant strain to show the ability of *PIPE-chipSAD* to handle experiments with different experimental designs.

## Materials and methods

*PIPE-chipSAD* was designed to investigate two color microarray datasets, but it is suitable to analyse data from different sources (one color microarray or sequencing technologies such as RNA-seq) with minimal data elaboration.

**Figure 1:**
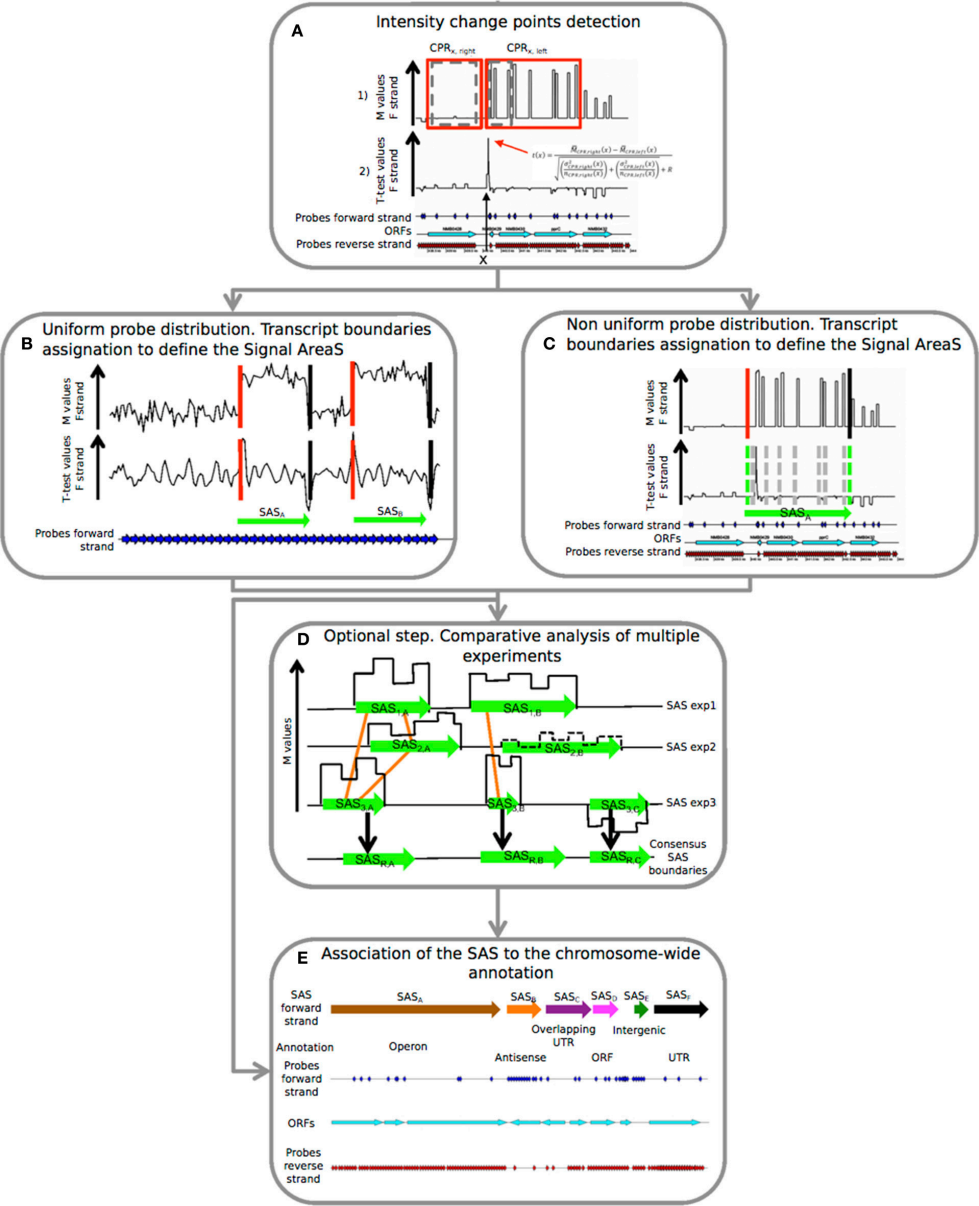
**Details of the five consecutive steps of the *PIPE-chipSAD*. *chipSAD* algorithm steps: (A)** In the first step, one pair of sliding and expanding windows is used to create two regions grouping consecutive probes of correlated signal intensity, named Correlated Probe Regions (CPRs). Pairs of consecutive CPRs are compared in the second step, using a modified *t*-test, to identify the positions in which the signal changes significantly. In **(B,C)** the boundaries of the signal areas (SAS) are determined for tiling and non-tiling probes design respectively, i.e., portion of the chromosome whose probes show similar intensity. This step provides a list of SAS boundaries. ***Align-chipSAD***: **(D)** This step is optional but necessary in case of a comparative analysis of multiple experiments. The result of this step is a unique list of SAS boundaries for several experiments, instead of one list for each experiment. ***Anno-chipSAD*****: (E)** The last step deals with the association of the SAS with the chromosome-wide annotation, Basically, it compares the identified SAS with the gbk file of the organism of interest.

The pipeline is composed by five steps, as indicated in the flow chart shown in Figure 1, that correspond to three main programs. This module structure provides more flexibility in the data analysis because the user can access to the different programs independently. The pipeline is freely available at: https://github.com/silviamicroarray/chipSAD.

### Identification of transcriptional units

*chipSAD* is the first program of the pipeline. The main algorithm defines pairs of contiguous sliding windows along the genomic coordinate *x*. The width of the windows is iteratively increased to include contiguous probes with a consistent signal. Finally, two correlated probe regions (CPRs) of different sizes are positioned at the two sides of each coordinate on the genome (Figure 1A in set 1 (red boxes)).

Then, it follows the identification of positions where the M value signal is subject to an abrupt change of intensity. A generalized *t*(*x*) parameter to compare the right and left CPR was defined as in (Tusher et al., 2001) in order to establish the signal “change points.” To create a signal area (SAS) a strategy based on the intensity value of contiguous CPRs was employed (Figures 1B,C). In case of tiling probes design, the evaluation of the *t*-test curve is enough to assess the starts and the ends of the SAS, as shown in Figure 1B. On the contrary, in case of non-tiling arrays (i.e., non uniform density of probes) shown in Figure 1C, the analysis of the *t*-test curve is not sufficient to provide good boundaries assessment thus the procedure is enriched with a criterion based on the M value comparison and a signal smoothing calculation based on the pseudomedian or Hodges-Lehmann estimator (Royce et al., 2007). Briefly, the averaged intensity of two regions, each limited by two consecutive change points, was compared: if their pseudomedian intensity was greater than the absolute value of the difference between the pseudomedian intensities of the two regions, they were joined and the comparison would extend to the next region, otherwise two different SAS were created. The result of this step is the list of SAS to be interpreted as putative transcripts.

### Comparative analysis of multiple experiments

In case of multiple experiments, *align-chipSAD* can be run. In order to avoid that noisy areas may influence the results of this procedure, only the SAS with a minimum value of the M pseudomedian ($\bar{\bar{M}}$) might be selected before running *align-chipSAD*. Then, graphs based on the overlap of the SAS are built as indicated in Figure 1D by the orange links. For each connected graph, the consensus boundaries are calculated considering a weighted average of the boundaries of the SAS belonging to the graph. In detail, the weighted position 〈*x*〉 (start or end) of the consensus SAS is:

$$\langle x\rangle =\frac{\sum_{i}p_{i}x_{i}}{\sum_{i}p_{i}}\text{ where }p_{i}=\frac{{\bar{\bar{M}}}_{i}}{\sum_{i}{\bar{\bar{M}}}_{i}}$$

with *i* = 1, 2…, *N* enumerates each SAS belonging to the graph.

Thus, for each connected graph, a consensus SAS is determined, and the conclusion of this step is a unique list of consensus SAS.

### Putative transcripts annotation

The final step of the *PIPE-chipSAD* is the comparison of the detected SAS with the genome annotation and the classification of putative transcripts when they represent single open reading frames (ORFs), entire operons (polycistronic transcripts), antisense RNAs, untranslated transcribed regions (UTRs) and intergenic RNAs (Figure 1E). This procedure consists in the comparison of the identified SAS with the gbk file of the organism of interest and is implemented by *anno-chipSAD*; comparing the identified SAS with the gbk file of the organism of interest. The first step of the classification was the selection of SAS overlapping annotated ORFs: if the SAS overlapped a minimum of 30% of a single ORF length in the same strand then it was classified as ORF (magenta arrow in Figure 1E), otherwise as antisense (orange arrow in Figure 1E). If the SAS overlapped two or more co-oriented ORFs in the sense strand (with a minimum of 30% of each ORF length), then it was classified as operon (brown arrow in Figure 1E). If the SAS overlapped two differently oriented ORFs and the intergenic region between the two ORFs was less than 30 bp, then it was classified as overlapping UTR (purple arrow in Figure 1E). If the SAS did not overlap an ORF in both strands and its length was less than 800 bases, it was classified as intergenic RNA (dark green arrow in Figure 1E). Finally, if the SAS overlapped both an ORF and the flanking intergenic region, at 5′ or 3′ ends, then it was classified as UTR (5′ or 3′ respectively) only if the gap between the ORF and the intergenic region is less than 30 bases (black arrow in Figure 1E).

### Datasets

We analyzed two previously published datasets used to study the transcriptome variation of *N. meningitidis* Δ*hfq* mutant strain with respect to wild type. The transcriptome data of the *N. meningitidis* MC58 *hfq* null mutant (Δ*hfq*) strain grown in GC medium described by Fagnocchi et al. (Fagnocchi et al., 2015) and the dataset presented by Mellin et al. (Mellin et al., 2010), which examined the *N. meningitidis* MC58 *hfq* null mutant and the relative complemented strain, grown in iron depleted (100 μg desferal ml^−1^) or replete conditions (100 μg ferric nitrate ml^−1^), were used in this study, to test the capability of *chipSAD* to handle data from different experimental designs. The microarrays analyzed are one or two-color hybridizations. In case of two-color experiments (Fagnocchi et al., 2015) a competitive hybridization between the null mutant and the wild type strains growth under the same *in vitro* growth condition was performed. In case of one-color experiments (Mellin et al., 2010) an *in silico* comparison to wild type strains growth was composed during global normalization. Three biological replicates of each experimental condition were available in each dataset. In order to make the data comparable, we merged the replicas of the same condition by averaging the M value of each probe after a global normalization of signals by using *limma* R package (Smyth and Speed, 2003).

### Parameter estimation

Automatic parameter estimation was done by the analysis of the distribution of probe M values. The determination of a suitable initial window size *w* was constrained by the spacing of the probes along the chromosome. Thus, the window size *w* was estimated calculating the average distance between consecutive probes. The parameter *m(w)* was estimated by calculating the minimum number of probes found in a window with size *w*.

## Results and discussion

### Comparative analysis of the transcriptome of *N. meningitidis* Δ*hfq* mutant in different growth conditions

**Figure 2:**
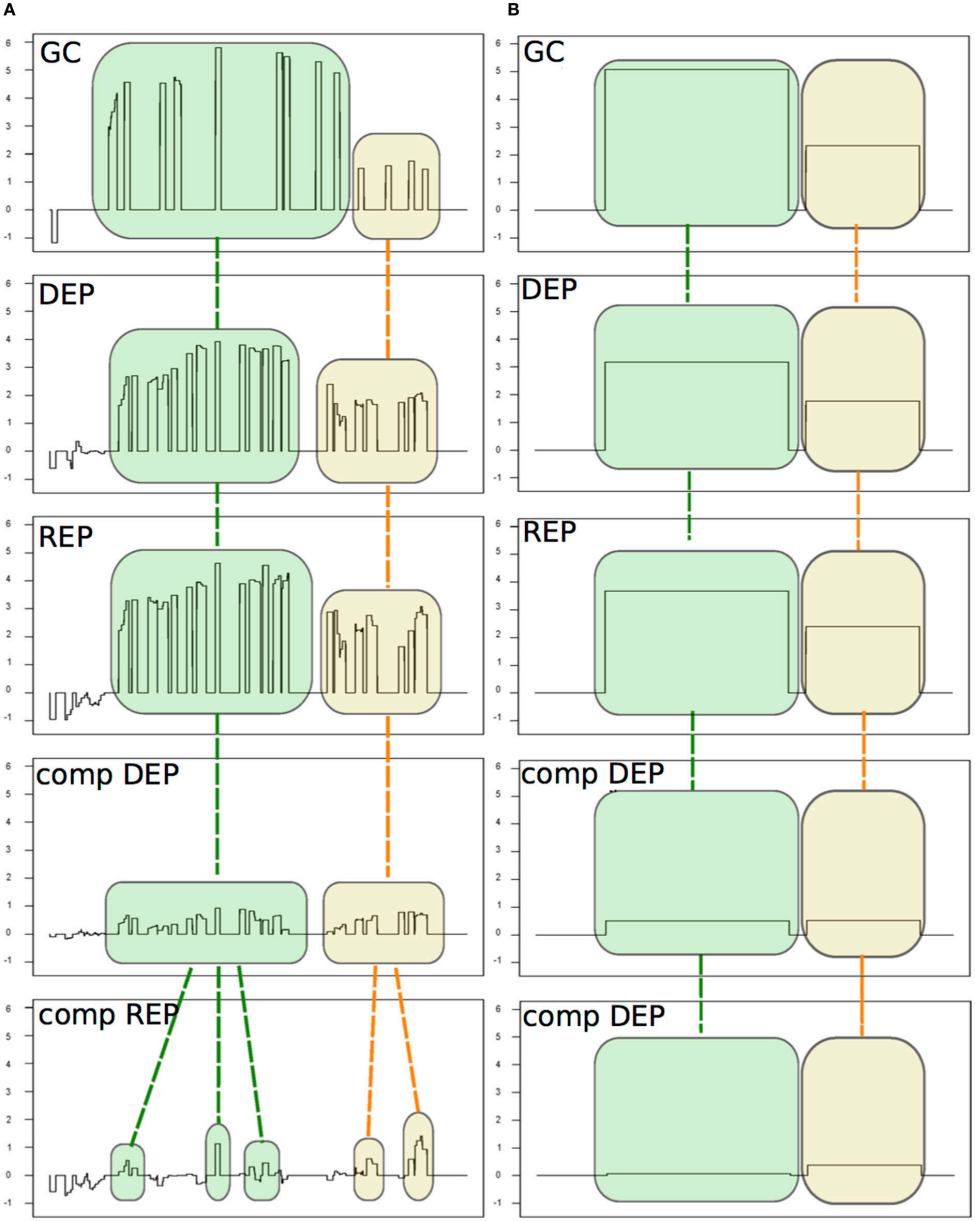
**An example of the alignment procedure of the detected signal areas (SAS)**. The five experiments are: Δ*hfq* mutant growth in GC medium (GC, from Fagnocchi et al., 2015) and four experiments from Mellin et al. (2010): the Δ*hfq* mutant and the complemented mutant (comp) grown under iron depleted (DEP) or replete (REP) conditions. **(A)** The SAS identified by *chipSAD* for the five experiments before the run of the alignment procedure. **(B)** The aligned SAS.

We analyzed two previously published datasets (Mellin et al., 2010; Fagnocchi et al., 2015) used to study the transcriptome variation of *Neisseria meningitidis* Δ*hfq* mutant strain to test the capabilities of *chipSAD* to manage different chip designs and hybridizations in a single analysis (Figure 2).

In Table 1 SM is reported the number of differentially expressed transcripts for each genomic category found by *PIPE-chipSAD* after the alignment of the two datasets. The application of *PIPE-chipSAD* allowed the detection of differentially expressed transcripts, especially for transcripts located in intergenic regions, in the five samples without affecting the results obtained running *chipSAD* on a single sample. In order to see how the alignment procedure, implemented in *align-chipSAD*, affects the SAS identified by *chipSAD*, we independently ran the *chipSAD* algorithm only on the Δ*hfq* mutant experiment of Fagnocchi's dataset and on the all experiments together and we compared the results. Among the 114 regions classified as single ORFs detected in the single run, 104 are still present after the alignment: 84 of those maintain the same classification, while 20 regions are rearranged in different kind of transcripts mainly as mRNA with UTRs or operons. Ten transcripts are no longer detected as differentially expressed after the application of *align-chipSAD*. A very similar scenario is observed with operons and mRNAs with UTRs. The 93 mRNAs with UTRs identified in the analysis of a single experiment overlap 115 transcripts (some mRNAs with UTRs comprise two genes), 74 of them are still classified as mRNAs with UTRs after the alignment procedure, while 33 transcripts are classified in a different class, more often in single ORFs than in operons. Finally, 100 out of 123 transcripts classified as operons belong to the same classification with the single run and upon *align-chipSAD* application. The intergenic regions are the most affected by the alignment procedure, because these regions usually are short and noisy. One hundred twenty-six intergenic regions out of 177 survive to the alignment of which 32 are joined in longer transcripts such as mRNA with UTR or operon. Twenty-seven intergenic regions are no longer present mainly because they had M values close to the threshold. Therefore, the main effect of the alignment procedure is the rearrangement of the regions in different classes respect to the classification obtained applying *chipSAD* on a single experiment. Anyway, after the alignment, only a small percentage of the transcripts have a class change, meaning that the boundaries of the regions detected by *chipSAD*, even considering only one experiment, are highly conserved. Moreover, only few transcripts are no longer detected after the alignment and most of them had very low M value, meaning that the *align-chipSAD* can improve the detection of significant regions removing noisy regions.

**Figure 3:**
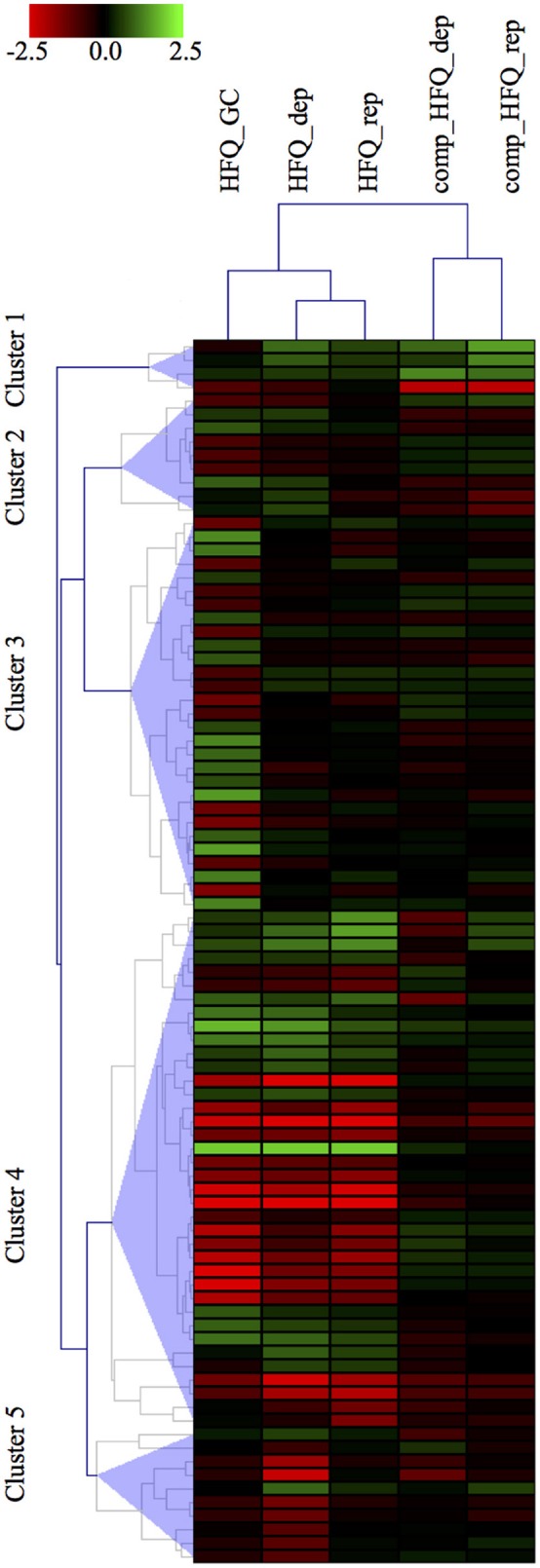
**Heatmap visualization of the Hfq-modulated transcripts**. Heatmap visualization of the top Hfq-modulated transcripts according to their differential expression vs. the wild type strain in each microarray experiment. The transcripts comprise of 35 ORFs, 28 operons, 10 UTRs, 17 intergenic regions.

In order to compare our results with the panel of 132 genes differentially expressed already published in Mellin et al. (Mellin et al., 2010), we first selected the transcripts specifically differentially expressed in the Δ*hfq* mutants with respect to both the wild type and also the complemented strain. Thus, we set up a threshold on the M value, selecting only those transcripts with a minimal expression fold change of 2 between the Δ*hfq* or the complemented vs wild type strain, in each growth condition. This selection criterion led to the identification of 90 transcripts, including 35 ORFs, 28 operons, 10 mRNA with UTRs, 17 intergenic regions (Figure 3), that are the most reliably and consistently Hfq-modulated and iron-dependent transcripts. Noteworthy, we found that 42 genes were identified by both approaches, however *PIPE-chipSAD* was able to determine whether these transcripts were organized in more complex structures such as operons or mRNA with UTRs. Furthermore, we individuated 31 previously unidentified Hfq-modulated and iron-dependent transcripts. Finally, our approach found 17 new intergenic regions to be specifically deregulated under iron starvation/abundance and in absence of Hfq, providing also the specific boundaries of the putative transcripts. Overall our method allowed a better understanding of the Hfq targetome. Finally, the results obtained by the application of *align-chipSAD* made the comparison of transcripts identified across datasets much easier, allowing a compact view such as a heat map (Figure 3).

We have presented *PIPE-chipSAD*, a specifically designed pipeline for bacterial transcriptomic analysis of high-density array data. The workflow is articulated in three main programs: *chipSAD, align-chipSAD* and *anno-chipSAD*. We previously used *chipSAD*, to analyse the transcriptome variation of *N. meningitidis* in a time course adaptation to human blood (Del Tordello et al., 2012) and in response to physiologically relevant growth conditions (Fagnocchi et al., 2015). These two successfully applications showed the widely applicability of this tool and the reliability of the achieved results. Here, the method was improved to achieve better performances in the segmentation of the intensity signal for both uniform and not uniform probe array designs (i.e., tiling and non-tiling microarray design). Moreover, we have showed that *align-chipSAD* was able to manage several experiments, with different experimental design, analyzing them simultaneously. Furthermore, we have demonstrated that *PIPE-chipSAD* allows tracing and studying the transcriptional profile of both coding and non-coding portions of the chromosome in a single framework. Overall, bearing the versatile characteristics of *PIPE-chipSAD*, we believe that it will be of wide applicability and it might be easily applied to analyse data from other high-throughput technologies such as RNA-seq.

## Author contributions

SB and AM designed and conceived the study. SB developed the software and analyzed the data. SB, ED, LF, CD, and AM contributed to development of the analysis tool and to results interpretation. SB and AM wrote the manuscript. All authors contributed, provided comments and approved the final manuscript.

## Funding

SB was recipient of a Novartis Vaccines fellowship from the Ph.D. program of the University of Siena. LF and ED were recipient of a Novartis Vaccines fellowship from the Ph.D. program of the University of Bologna. The author AM was employed by the funding organization, Novartis Vaccines now a GSK company.

### Conflict of interest statement

The authors declare that the research was conducted in the absence of any commercial or financial relationships that could be construed as a potential conflict of interest.

## Abbreviations

CPR: correlated probe region, *ie* clusters of contiguous probes having a correlated signal
SAS: signal areas, *ie* putative transcribed regions
ORF: open reading frame
UTR: untranslated transcribed region.
