# Supplementary material for: PIPE-chipSAD: A Pipeline for the Analysis of High Density Arrays of Bacterial Transcriptomes
Source: Front Mol Biosci. 2016 Dec 20;3:82. doi: 10.3389/fmolb.2016.00082 (PMC5167695; doi:10.3389/fmolb.2016.00082)
Supplement: Supplementary file 1 [file Table1.DOCX]

# Supplemental Information

| **Transcript class** | **(a) Number of DE transcripts identified in the experiment *Δhfq* growth in GC (**[**Fagnocchi, et al.**](#_ENREF_1)**)** | **(b) Number of DE transcripts after the alignment of the 5 *Δ hfq* experiments (**[**Fagnocchi, et al.**](#_ENREF_1)**;** [**Mellin, et al., 2010**](#_ENREF_2)**)** | **(c) Number of DE transcripts (a) that belong to the same category before and after the alignment** | **(d) Number of DE transcripts (a) that belong to a different category after the alignment** | **(e) Number of DE transcripts (a) that are no more identified after the alignment** |
| --- | --- | --- | --- | --- | --- |
| ORF | 114 | 382 | 84 | 20 | 10 |
| Operon | 123 (transcripts) 363 (genes) | 188 (transcripts) 493 (genes) | 100 (transcripts, 66 of which have the same organization) 267 (genes) | 27 | 69 |
| mRNA with UTRs | 93 (transcripts) 115 (genes) | 118 (transcripts)  170 (genes) | 74 genes | 33 | 6 |
| small intergenic RNA | 177 | 314 | 94 | 32 | 27 |
| Total | 507 | 1002 | 352 | 112 | 112 |

**Table 1 SM.** Comparison of the differentially expressed transcripts identified in the *N. meningitidis* Hfq mutant growth in GC medium ([Fagnocchi, et al.](#_ENREF_1), 2015) and the transcripts identified in the whole dataset using the *align-chipSAD*. Column (a): number of differentially expressed (DE) transcripts for each class identified running *chipSAD* on the single *hfq* mutant growth in GC medium ([Fagnocchi, et al.](#_ENREF_1) , 2015). Column (b): number of DE transcripts for each class identified running *align-chipSAD* on the five experiments (1 from Fagnocchi’s dataset ([Fagnocchi, et al.](#_ENREF_1) , 2015) and 4 from Mellin’s dataset ([Mellin, et al., 2010](#_ENREF_2))). Columns (c-e) report the number of transcripts from column (a) identified for each class as result of the alignment on the 5 experiments. Since the chip layout of Mellin *et al.* is lacking of antisense probes the analysis for this kind of transcripts was not included.
